# Which value aspects are relevant for the evaluation of medical devices? Exploring stakeholders’ views through a Web-Delphi process

**DOI:** 10.1186/s12913-023-09550-0

**Published:** 2023-06-08

**Authors:** Liliana Freitas, Ana C. L. Vieira, Mónica D. Oliveira, Helena Monteiro, Carlos A. Bana e Costa

**Affiliations:** 1 CEG-IST, Instituto Superior Técnico, Universidade de Lisboa, Av. Rovisco Pais, 1049-001 Lisbon, Portugal; 2 iBB- Institute for Bioengineering and Biosciences and i4HB- Associate Laboratory Institute for Health and Bioeconomy, Instituto Superior Técnico, Universidade de Lisboa, Av. Rovisco Pais, 1, Lisbon, 1049-001 Portugal; 3 Infarmed, I. P., Lisbon, Portugal; 4 LSE Health-Medical Technology Research Group (MTRG), London School of Economics, Houghton St, London, WC2A 2AE UK

**Keywords:** Medical devices, Health technology assessment, Web-Delphi, Health stakeholders, Implantable medical devices, In vitro tests based on biomarkers

## Abstract

**Background:**

Implementation and uptake of health technology assessment for evaluating medical devices require including aspects that different stakeholders consider relevant, beyond cost and effectiveness. However, the involvement of stakeholders in sharing their views still needs to be improved.

**Objective:**

This article explores the relevance of distinct value aspects for evaluating different types of medical devices according to stakeholders' views.

**Methods:**

Thirty-four value aspects collected through literature review and expert validation were the input for a 2-round Web-Delphi process. In the Web-Delphi, a panel of participants from five stakeholders’ groups (healthcare professionals, buyers and policymakers, academics, industry, and patients and citizens) judged the relevance of each aspect, by assigning a relevance-level (‘Critical’, ‘Fundamental’, ‘Complementary’, or ‘Irrelevant’), for two types of medical devices separately: ‘Implantable’ and ‘In vitro tests based on biomarkers’. Opinions were analysed at the panel and group level, and similarities across devices were identified.

**Results:**

One hundred thirty-four participants completed the process. No aspects were considered ‘Irrelevant’, neither for the panel nor for stakeholder groups, in both types of devices. The panel considered effectiveness and safety-related aspects ‘Critical’ (e.g., ‘Adverse events for the patient’), and costs-related aspects ‘Fundamental’ (e.g., ‘Cost of the medical device’). Several additional aspects not included in existing frameworks’ literature, e.g., related to environmental impact and devices’ usage by the healthcare professional, were deemed as relevant by the panel. A moderate to substantial agreement across and within groups was observed.

**Conclusion:**

Different stakeholders agree on the relevance of including multiple aspects in medical devices’ evaluation. This study produces key information to inform the development of frameworks for valuing medical devices, and to guide evidence collection.

**Supplementary Information:**

The online version contains supplementary material available at 10.1186/s12913-023-09550-0.

## Background

Health Technology Assessment (HTA) allows to appraise the relative value of health technologies, to support their introduction and use in healthcare systems, and to inform other healthcare decisions [1–3], such as reimbursement, coverage and pricing [4, 5]. Among health technologies, medicines have been the traditional focus of HTA studies, with HTA agencies often relying on economic evaluation studies (mainly on cost-effectiveness analysis) to inform their decisions [6, 7]. Nevertheless, increasing attention is being given to whether costs and effectiveness are the only relevant aspects to capture health technology’s value, namely for evaluating medical devices [1, 3, 4, 8–10].

Medical devices cover a wide range of health technologies, from assistive devices to sophisticated implants, and can facilitate disease prevention, diagnosis, and treatment [11]. The World Health Organization estimates that there are 2 million different medical devices [12]. The diversity and rapid pace of innovation of this industry, together with the recent European regulations [13–15], highlight the need to systematise the HTA process for these types of technologies [4, 9]. When comparing to HTA processes for drugs, researchers highlight undeniable differences that can be brought by the device-specific features [1, 16, 17]. Specifically, the device-operator interaction, the incremental and rapid innovation, or the difficulty in conducting randomised controlled trials to produce high-quality evidence have been features recalled to impact on evidence and on evaluations in practice [3, 7, 18].

Exploring how the evaluation of medical devices is being conducted in Europe, Fuchs et al. [3] interviewed 16 representatives from HTA institutions and observed differences across and within countries in the value aspects considered when evaluating medical devices, with aspects depending on its relevance for evaluators and on the type and purpose of the devices being assessed. These findings relate to a lack of methodological guidance in the area, as pointed out by Ciani et al. [7] when analysing practices across 36 non-European HTA agencies.

Researchers have been attempting to develop evaluation frameworks to standardise and bring guidance and transparency to the evaluation of health technologies, including medical devices [19–21]. Nevertheless, a recent systematic review [22] describing HTA-related value assessment frameworks showed that there is still no consensus on how to define health technology’s value with the frameworks differing in the value aspects included. Furthermore, several authors acknowledge that these frameworks fail to consider a wide range and diversity of stakeholders’ perspectives [19], with the engagement of patients and public being very limited or missing [22, 23]. The inclusion of stakeholders’ views is described as key to enable HTA adoption [24–26], with Mueller et al. [27] concluding – about the importance of involving stakeholders in HTA – that “engaging and consulting stakeholders locally was imperative to understand the context, reduce evidence gaps and address the uncertainties in the evidence, ultimately paving the way for technology adoption” (p.14).

Several of those frameworks have explored the use of multicriteria decision analysis (MCDA) as a frame both to consider multiple evaluation aspects, explicitly and transparently, and to include stakeholders’ views within evaluations [2, 24]. Nevertheless, the difficulties in synthesizing key information and in establishing evaluation criteria, and the fact that MCDA modelling usually rely on small numbers of participants, have been pointed out as shortcomings [2, 28]. To overcome these shortcomings, Web-Delphi processes have been used in other health contexts to gather stakeholders’ opinions, promote discussions or consensus [29, 30], and to produce information for assisting MCDA modelling [2, 31, 32]. These processes have shown to gather opinions from large and heterogeneous groups, to promote consensus and to generate a collaborative environment, under a low cost format [33]. This article aims to explore the views of different stakeholders regarding the relevance of a set of value aspects for evaluating two distinct types of medical devices, ‘Implantable medical devices’ and ‘In vitro tests based on biomarkers’, through a Web-Delphi process. Results from this process can inform HTA processes in practice, discussions about which value aspects are relevant to consider in medical devices evaluation frameworks (including in MCDA-based frameworks), as well as the design of tools for evaluating distinct types of devices.

## Methods

### Overview of the Web-Delphi process

This study was developed within the scope of the MEDI-VALUE (“Developing HTA tools to consensualise MEDIcal devices’ VALUE through multicriteria decision analysis”) project [34], a national research project that included as consortium partners the Portuguese national HTA agency (INFARMED) and three leading Portuguese hospitals (Centro Hospitalar Lisboa Norte, Hospital do Espírito Santo de Évora and Instituto Português de Oncologia de Lisboa), and aimed at advancing HTA literature by designing and implementing methods, informed by MCDA, that enable the involvement of a large number of health stakeholders and promote consensus in the structuring and development of sound models for assessing the multidimensional value of medical devices.

**Fig. 1:**
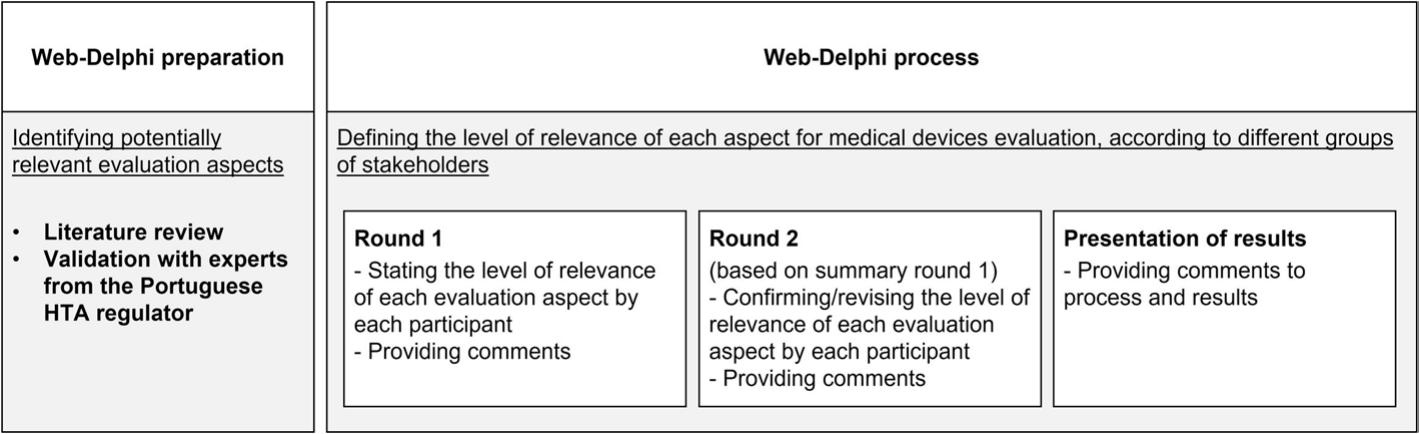
Design of the Web-Delphi process

To inform HTA processes and to inform the building of evaluation models, this study presents a Web-Delphi process designed (see Fig. 1) for gathering different stakeholders’ opinions on the relevance of distinct aspects for medical devices’ evaluation in terms of their added-value to an alternative comparator. Delphi processes allow to anonymously collect individual opinions in successive rounds presenting, from round 2 onwards, a summary of the opinions given in the previous round to participants [35]. This design allows participants to reflect on their previous opinions and change them, or not, based upon new generated information.

In preparation for this process, the aspects potentially relevant for the evaluation of medical devices were collected from studies using MCDA for medical devices evaluation, identified in the systematic review of Oliveira et al. [2], and by extending the search protocol of that study until October of 2019. From this search, a final list of 34 aspects (described in Additional file 1) was organised, with two MEDI-VALUE experts from INFARMED (the Portuguese HTA regulator) analysing the list completeness, eliminating evident redundancies.

The Web-Delphi was implemented in the Welphi platform [36] and was composed of two rounds. In the first round (12 March-02 May 2020), participants were invited to give their opinion about the relevance of each of the 34 aspects for each of the two types of medical devices, by choosing one level of the four-level qualitative relevance scale presented below. This relevance scale allows not only to identify which aspects are relevant for stakeholders, but also to capture the strength of the relevance, either for the panel or for each stakeholder group. Following the rational of “determinants” [37] and “relevancy” [38] analysis (which is context specific [39]), this information is useful for informing the structuring of a multicriteria value framework, as it helps in screening out non-relevant aspects, and for assisting the building of multicriteria evaluation models, as it differentiates the relevance among aspects. The scale was developed, tested and validated with five health experts from MEDI-VALUE partners.

- Critical: this aspect, beyond fundamental, can, by itself, preclude assessing if the medical device has added value given its alternative.
- Fundamental: this aspect must, undoubtedly, be part of the basis of evaluation of the medical device, to assess if it has added value given its alternative.
- Complementary: this aspect is not fundamental but, still, it can add something to the value of the medical device given its alternative.
- Irrelevant: this aspect must not be part of the basis of evaluation of the medical device; it is inapplicable or irrelative to assess if the medical device has added value given its alternative.

The 34 aspects and the relevance scale were presented to participants in two separate screens: for ‘Implantable medical devices’ and for ‘In vitro tests based on biomarkers’ (a therapeutic and a diagnostic type of device, respectively). For each type of medical device, participants could provide general comments and, for each aspect individually, they could select the ‘Don’t know/ Don’t want to answer’ option or provide specific comments. Participants could give their opinions for one or both types of medical devices. In the second round (9–31 May 2020), participants had access to similar screens, but then they could access their own previous answers, and the distribution of all first-round answers and comments as feedback, being able to change their answer. After the second round was concluded, participants could access the results and leave comments regarding the results and the process (23 June-14 July 2020). A second study was developed simultaneously, exploring the effect of feeding back the distribution of the answers disaggregated per groups of stakeholders on participants’ opinion change. For this, half of the participants were randomly selected to also have access to such information. A manuscript of this second study is being prepared, and more information can be provided upon request.

### Invited participants

Participants’ selection followed a purposive sampling strategy targeting five stakeholders’ groups in Portugal: healthcare professionals (doctors, nurses, pharmacists, technicians), buyers and policymakers, academics, industry, and patients and citizens. MEDI-VALUE partners disseminated the study among their networks, explaining the study's aim and the type of participants being recruited. Specifically, selected stakeholders should: (a) have experience in the use, evaluation, selection or acquisition of medical devices, or having an interest in the topic; (b) be available to participate given the timeline planned; (c) not have any conflict of interest preventing their impartial participation. Through this process, a total of 365 stakeholders were identified and invited to participate. As each participant can have several roles in practice and, thus, could belong to more than one group, participants selected, at the beginning of the Web-Delphi, the stakeholder group in the light of which they would give their opinions.

### Analysis

Analyses were performed in Microsoft Excel and R software to answer three specific research questions: to explore the relevance of each aspect for the evaluation of the two types of medical devices, for the panel and per stakeholders’ groups; to measure the level of agreement within groups; and to explore whether there were aspects with the same relevance for the two types of medical devices.

#### Relevance of each aspect for the evaluation of the two types of medical devices

For each type of medical device, the answers were analysed at the panel-level and, then, at the stakeholders’ groups-level. A panel majority opinion about each aspect's relevance was defined at the end of the process, based upon the absolute majority of answers (more than 50%). Specifically, if there was a relevance level gathering absolute majority, it would be selected to describe the panel majority opinion, otherwise panel majority opinion would be described as ‘No majority’. The distribution of answers per group of stakeholders was analysed to draw conclusions on how aligned the groups were with the panel, in each aspect. To complement this analysis, the Kruskal–Wallis H-test [40], with 4 degrees of freedom, was used to explore whether the results were significantly different across the distinct groups. As this test only reveals if there is a difference and does not specify between which groups the differences occur, the post-hoc Dunn’s test was performed, corrected with the Bonferroni test, for the cases in which the Kruskal–Wallis test found significant differences [40]. Differences across stakeholders’ groups were considered significant for Dunn’s-Bonferroni p-value lower than 0.05.

#### Agreement within groups of stakeholders for the two types of medical devices

The Gwet’s AC2 agreement coefficient [41, 42] was used to determine the inter-rater reliability within each group of stakeholders, with the quadratic weighting scheme [43] used to compute the coefficient. Afterwards, Gwet’s coefficients were compared to the Landis and Koch benchmark levels [44] that establish the agreement as poor (for coefficient values < 0), slight (0.00–0.20), fair (0.21–0.40), moderate (0.41–0.60), substantial (0.61–0.80) or almost perfect (0.81–1), This comparison allowed us to determine the strength of agreement according to the Landis and Koch scale [44]. Despite the high support of this benchmarking scale among researchers [45], Gwet [41] points out that the AC coefficients have a probability distribution and an error margin associated with them, and that the benchmarking approach should account for that uncertainty. Thus, we have also applied Gwet’s proposed benchmarking method [41] that determines the agreement level (of a selected benchmarking scale) associated with a 95% confidence. We have adopted the Landis and Koch benchmark scale, due to its finer categorization [41], and considered the 95% cut-off point for the cumulative probabilities. As this benchmarking method considers the standard error of the computed coefficients, the choice of the agreement level can be more conservative than applying Landis and Koch benchmark alone [41].

#### Common relevance for both types of medical devices

Finally, it was explored whether similar conclusions about the relevance of each aspect could be retrieved for both types of medical devices simultaneously. For that purpose, the panel and the groups majority opinions were compared, and the aspects gathering the same relevance in both types of medical devices were identified.

## Results

Web-Delphi’s first round was completed by 167 participants, with 134 (80.2%) completing the second round. The distribution of participants per group of stakeholders and the dropout rates are presented in Table 1.

**Table 1:** Participants in the Web-Delphi process, per group of stakeholders, and dropout rate

Groups of stakeholders	Round 1			Round 2			Dropout (%)
	Implantable medical devices	In vitro tests based on biomarkers	**Total**	Implantable medical devices	In vitro tests based on biomarkers	**Total**	
Academics	26	25	27	22	21	23	14,8%
Buyers and policymakers	10	8	10	8	6	8	20,0%
Healthcare professionals (doctors, nurses, pharmacists, technicians)	69	65	74	52	48	57	23,0%
Industry	16	15	17	12	11	13	23,5%
Patients and citizens	39	39	39	33	33	33	15,4%
**TOTAL**	160	152	167	127	119	134	19,8%

Table 1 also details how many participants concluded the Web-Delphi for ‘implantable’ devices (127) and for ‘in vitro’ devices (119). These numbers of participants were used in the following analyses, as each type of medical of devices is looked at individually.

### Relevance of each aspect for the evaluation of each of the two types of medical devices

Table 2 presents the distribution of answers, per type of medical device, of the panel and disaggregated per group of stakeholders. Moreover, it presents the panel majority opinion and the analysis on whether the groups are aligned with the panel, described as the groups majority opinions.

**Table 2:**
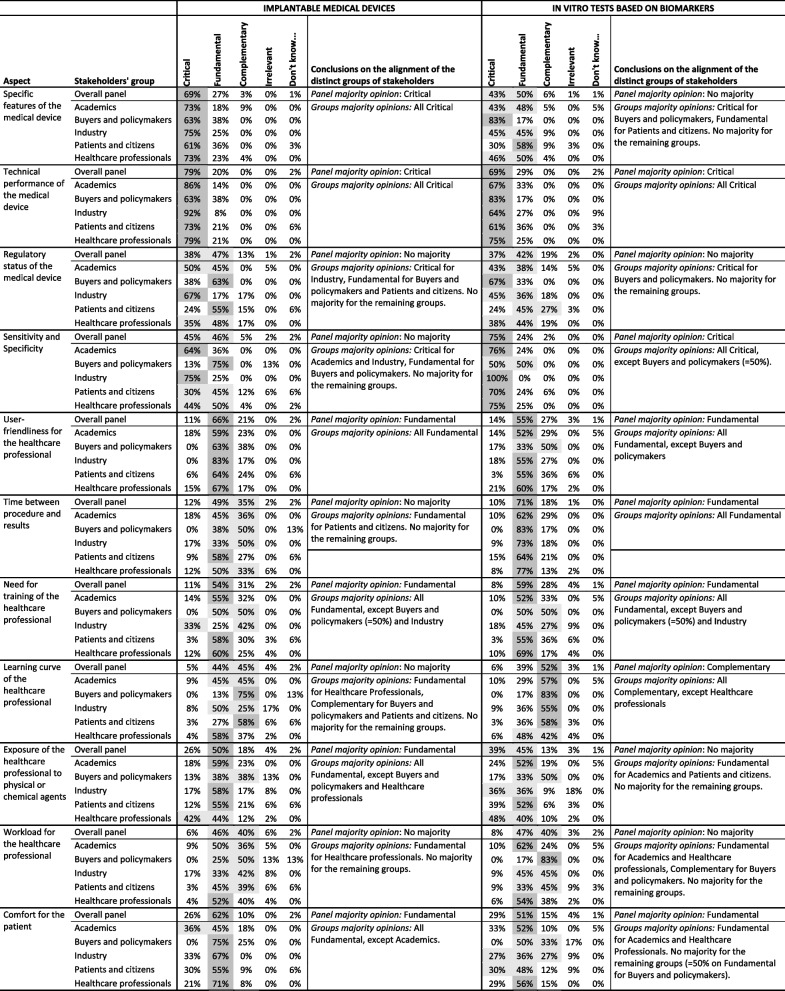

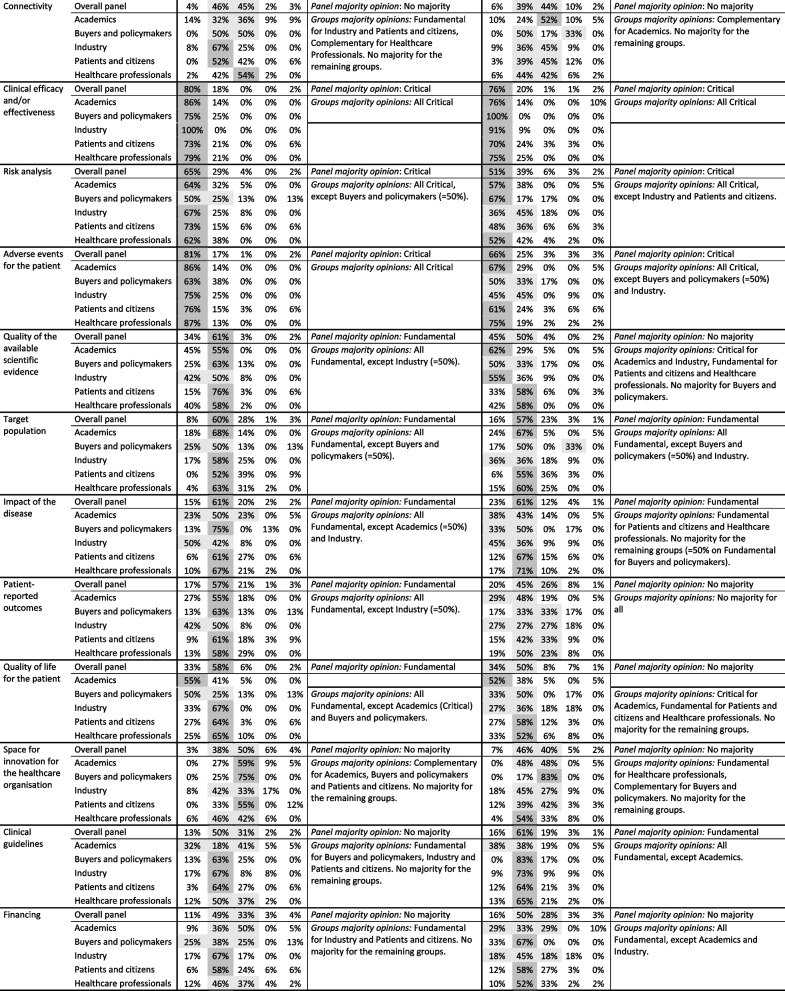

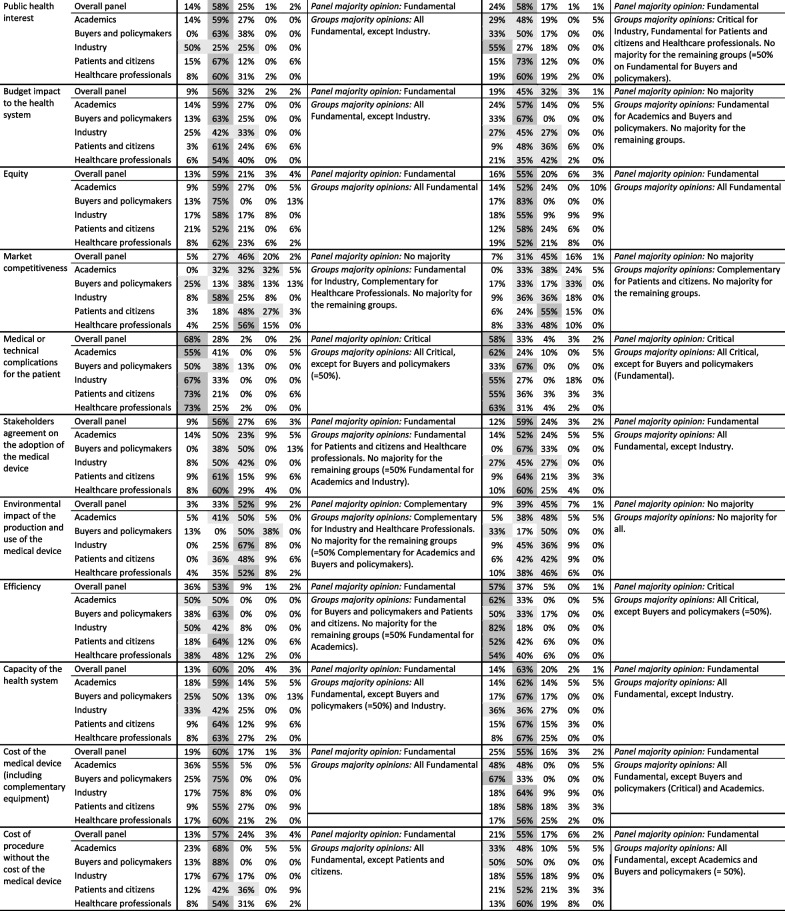
Aspects, distribution of answers, panel majority opinion and groups majority opinions

‘Don’t know…’ column present the answers for ‘Don’t know / Don’t want to answer’. The relevance levels gathering majority are highlighted in dark grey and, if there is no majority, the relevance levels gathering most answers are highlighted in light grey

For ‘Implantable medical devices’ six aspects gathered panel majority on ‘Critical’, 16 on ‘Fundamental’, and one on ‘Complementary’. Regarding ‘In vitro tests based on biomarkers’, seven aspects gathered panel majority on ‘Critical’, 13 on ‘Fundamental’ and one on ‘Complementary’. For both types of medical devices, the remaining aspects had no panel majority opinion. These results suggest that the panel considers all 34 aspects as relevant to evaluate the added value of a medical device for any of these types of devices – as there was no aspects gathering majority on ‘Irrelevant’ – and, specifically, that the panel agrees in the relevance of a high number of aspects – 23 and 21 aspects for ‘implantable’ and ‘in vitro’ devices, respectively.

When analysing the answers per groups of stakeholders, we observe some differences. For ‘Implantable medical devices’, for four of the six above-mentioned ‘Critical’ aspects and three of the 11 ‘Fundamental’ aspects, the majority occurred across all stakeholders’ groups, with the remaining aspects presenting one to three groups of stakeholders without the same majority opinion. Identically, for ‘In vitro tests based on biomarkers’, only two of the five ‘Critical’ aspects and two of the 11 ‘Fundamental’ aspects presented the same majority across all groups. Regarding the aspects with no panel majority opinion, most present a majority on one to three groups of stakeholders, with only two aspects (‘Patient-reported outcomes’ and ‘Environmental impact of the production and use of the medical device’) of ‘in vitro’ devices not gathering majority in any group.

In all cases, for the aspects with no groups majority opinions, the answers of the groups tend to be around the same two relevance levels (these two levels are highlighted in light grey in Table 2). Exceptions to this are, for example, ‘Exposure of the healthcare professional to physical or chemical agents’ that presents groups of stakeholders with most answers around ‘Critical’ and ‘Fundamental’ and other groups with most answers around ‘Fundamental’ and ‘Complementary’ (this happening for both types of medical devices, with a panel majority opinion defined only in ‘implantable’ devices).

For ‘Implantable medical devices’ a statistically significant difference (statistical tests results available in Additional file 2) was found between Industry and Patients and citizens in ‘Sensitivity and Specificity’ (*p* = 0.0449), for which Industry mostly considered ‘Critical’ and Patients and citizens had a dispersed opinion with no majority, and ‘Impact of the disease’ (*p* = 0.0282), for which Industry group’s opinion gathered no majority and Patients and citizens gathered majority in ‘Fundamental’; between Healthcare professionals and Patients and citizens in ‘Exposure of the healthcare professional to physical or chemical agents’ (*p *= 0.0405), for which Healthcare professionals gathered no majority and Patients and citizens gathered majority in ‘Fundamental’; between Academics and Patients and citizens in ‘Target population’ (*p* = 0.0447) and ‘Cost of the medical device (including complementary equipment)’ (*p* = 0.0266), in which both groups gathered majority on ‘Fundamental’ but Academics gathered opinion among ‘Critical’ and ‘Fundamental’, and Patients and citizens among ‘Fundamental’ and ‘Complementary’; and between Academics and Healthcare professionals in ‘Cost of procedure without the cost of the medical device’ (*p* = 0.0326), with both groups gathering majority in ‘Fundamental’ but Academics gathering opinion among ‘Critical’ and ‘Fundamental’ and Healthcare professionals among ‘Fundamental’ and ‘Complementary’. For ‘In vitro tests based on biomarkers’, a statistically significant difference was found between Academics and Patients and citizens in ‘Target population’ (*p* = 0.0370), with both groups gathering majority in ‘Fundamental’ but Academics gathering opinion among ‘Critical’ and ‘Fundamental’ and Patients and citizens among ‘Fundamental’ and ‘Complementary’, and between Academics and Healthcare professionals in ‘Cost of the medical device (including complementary equipment)’ (*p* = 0.00718), with Academics’ answers gathering no majority and Healthcare professionals gathering majority in ‘Fundamental’. For the remaining aspects, the differences across groups were not statistically significant.

### Agreement within groups of stakeholders for each of the two types of medical devices

All groups presented a higher Gwet’s AC2 coefficient for ‘implantable’ than for ‘in vitro’ devices (see Additional file 3). Irrespectively of the type of medical device, the agreement increased in round 2, suggesting answers’ convergence. According to both benchmarking methods, Landis and Koch [44] and Gwet’s proposed benchmarking method [41], the calculated Gwet’s coefficients describe a strength of agreement from ‘moderate’ to ‘substantial’ among all stakeholders’ groups. The agreement level assessed first, following Landis and Koch, and after, using Gwet’s proposed benchmarking method, comprised the following differences: for ‘Implantable medical devices’, the agreement changed from ‘substantial’ to ‘moderate’ in the Buyers and policymakers group (in round 1) and for ‘In vitro tests based on biomarkers’, the agreement changed from ‘substantial’ to ‘moderate’ in the Buyers and policymakers group (in round2). Both benchmarking methods resulted in a ‘moderate’ agreement in the Industry group (in both rounds for ‘in vitro’ devices) and in the Buyers and policymakers group (in round 1, for ‘in vitro’ devices). A ‘substantial’ agreement was observed in all the remaining cases.

### Common relevance for both types of medical devices

Similar results were retrieved for both types of medical devices: five aspects were ‘Critical’ and 11 ‘Fundamental’, according to the panel – in three ‘Critical’ and in one ‘Fundamental’, the majority was observed in all stakeholders’ groups. In the remaining 18 aspects, seven aspects did not gather majority in any type of device, 10 gathered majority for only one type and 1 gathered majority on a different relevance level across both types of devices. These results, previously detailed in Table 2, are synthesised in Table 3 together with the common relevance level.

**Table 3:** Summary of conclusions about the common relevance of aspects for both types of medical devices

Common relevance	Aspect	Implantable medical devices (127 participants)						In vitro diagnostic tests based on biomarkers (119 participants)					
		Panel majority opinion	Groups majority opinions (✓ if aligned with panel majority opinion)					Panel majority opinion	Groups majority opinions ( if aligned with panel majority opinion)				
			A	BP	I	PC	HP		A	BP	I	PC	HP
**Critical**	Technical performance of the medical device	Critical	✓	✓	✓	✓	✓	Critical	✓	✓	✓	✓	✓
	Clinical efficacy and/or effectiveness	Critical	✓	✓	✓	✓	✓	Critical	✓	✓	✓	✓	✓
	Risk analysis	Critical	✓		✓	✓	✓	Critical	✓	✓			✓
	Adverse events for the patient	Critical	✓	✓	✓	✓	✓	Critical	✓	✓	✓	✓	✓
	Medical or technical complications for the patient	Critical	✓		✓	✓	✓	Critical	✓	Fu	✓	✓	✓
**Fundamental**	User-friendliness for the healthcare professional	Fundamental	✓	✓	✓	✓	✓	Fundamental	✓		✓	✓	✓
	Need for training of the healthcare professional	Fundamental	✓			✓	✓	Fundamental	✓			✓	✓
	Comfort for the patient	Fundamental		✓	✓	✓	✓	Fundamental	✓				✓
	Target population	Fundamental	✓		✓	✓	✓	Fundamental	✓			✓	✓
	Impact of the disease	Fundamental		✓		✓	✓	Fundamental				✓	✓
	Public health interest	Fundamental	✓	✓		✓	✓	Fundamental			Cr	✓	✓
	Equity	Fundamental	✓	✓	✓	✓	✓	Fundamental	✓	✓	✓	✓	✓
	Stakeholders agreement on the adoption of the medical device	Fundamental				✓	✓	Fundamental	✓	✓		✓	✓
	Capacity of the health system	Fundamental	✓			✓	✓	Fundamental	✓	✓		✓	✓
	Cost of the medical device (including complementary equipment)	Fundamental	✓	✓	✓	✓	✓	Fundamental		Cr	✓	✓	✓
	Cost of procedure without the cost of the medical device	Fundamental	✓	✓	✓		✓	Fundamental			✓	✓	✓
**-**	Specific features of the medical device	Critical	✓	✓	✓	✓	✓	No majority		Cr		Fu	
	Regulatory status of the medical device	No majority		Fu	Cr	Fu		No majority		Cr			
	Sensitivity and Specificity	No majority	Cr	Fu	Cr			Critical	✓		✓	✓	✓
	Time between procedure and results	No majority					Fu	Fundamental	✓	✓	✓	✓	✓
	Learning curve of the healthcare professional	No majority		Co		Co	Fu	Complementary	✓	✓	✓	✓	
	Exposure of the healthcare professional to physical or chemical agents	Fundamental	✓		✓	✓		No majority	Fu			Fu	
	Workload for the healthcare professional	No majority					Fu	No majority	Fu	Co		Fu	
	Connectivity	No majority			Fu	Fu	Co	No majority	Co				
	Quality of the available scientific evidence	Fundamental	✓	✓		✓	✓	No majority	Cr		Cr	Fu	Fu
	Patient-reported outcomes	Fundamental	✓	✓		✓	✓	No majority					
	Quality of life for the patient	Fundamental	Cr		✓	✓	✓	No majority	Cr			Fu	Fu
	Space for innovation for the healthcare organization	No majority	Co	Co		Co		No majority		Co			Fu
	Clinical guidelines	No majority		Fu	Fu	Fu		Fundamental		✓	✓	✓	✓
	Financing	No majority			Fu	Fu		No majority		Fu		Fu	Fu
	Budget impact to the health system	Fundamental	✓	✓		✓	✓	No majority	Fu	Fu			
	Market competitiveness	No majority			Fu		Co	No majority				Co	
	Environmental impact of the production and use of the medical device	Complementary			✓		✓	No majority					
	Efficiency	Fundamental		✓		✓		Critical	✓		✓	✓	✓

*A* Academics, *BP* Buyers and policymakers, *I* Industry, *PC* Patients and citizens, *HP* Healthcare professionals, *Cr* Critical, *Fu* Fundamental, *Co* Complementary

## Discussion

Based upon the opinions of the distinct stakeholders’ groups involved in the Web-Delphi process, this study, firstly, explored the relevance of 34 aspects for the evaluation of each of two types of medical devices, according to the panel and per group of stakeholders; secondly, analysed the level of agreement within stakeholders’ groups; and, finally, concluded about which aspects gathered a common relevance across the two types of medical devices. This work was developed to form a basis for discussing HTA processes and the construction of value models to evaluate medical devices. It is hereinafter discussed in terms of stakeholders’ views on the aspects to consider in medical devices evaluation, implications for policy, and limitations of the study.

### Stakeholders’ views on the aspects to consider in medical devices evaluation

The Delphi panel was composed of distinct stakeholders’ groups. Analysing the distribution of answers of the panel and per groups, results show one of four situations: (1) there is a panel majority opinion with all groups presenting majority on the same relevance level, (2) there is a panel majority opinion but not all groups present majority on the same relevance level, (3) there is no panel majority opinion but some groups present majority on a relevance level, and (4) there is no panel majority opinion nor majority in any group. Considering the meaning of the relevance levels (presented in *Overview of the Web‑Delphi process*, in the *Methods *section), these results suggest that participants consider there are aspects that must always be part of the basis of evaluation: that is the case of aspects assigned ‘Fundamental’ or ‘Critical’ by the panel, for instance, ‘User-friendliness for the healthcare professional’ for ‘implantable’ devices and ‘Time between procedure and results’ for ‘in vitro’ devices. Furthermore, participants consider some of these aspects can even preclude the evaluation if there is no data for assessing them – this applies to aspects set as ‘Critical’ –, for instance, ‘Specific features of the medical device’ for ‘implantable’ devices and ‘Sensitivity and Specificity’ for ‘in vitro’ devices. Additionally, participants’ answers suggest that there are ‘Complementary’ aspects, i.e., aspects that can add some value but will not always be part of the basis of evaluation, for instance ‘Environmental impact of the production and use of the medical device’ and ‘Learning curve of the healthcare professional’ for ‘implantable’ and ‘in vitro’ devices, respectively. This can be seen in Table 3 that presents the panel majority opinion and systematizes the groups majorities aligned with the panel. In general, stakeholders’ groups did not present obvious contradictory opinions, as even the aspects with no panel majority opinion gathered most groups answers around the same two consecutive relevance levels (as presented in Table 2). The Kruskal–Wallis test followed by Dunn’s-Bonferroni post hoc method allowed to identify only eight aspects (six in ‘implantable’ and two in ‘in vitro’ devices) with statistically significant differences across groups, but these differences were always observed across only one pair of groups, and the inter-rater reliability calculated with Gwet’s AC2 agreement coefficient showed a strength of agreement from moderate to substantial within each group, suggesting an alignment of the panel and within groups. Despite the general alignment of the groups, the reasons underlying the observed differences may be of interest for further research [46].

This Web-Delphi process collected opinions not only from different stakeholders’ groups but also for two types of devices, a therapeutic and a diagnostic type of device. Comparing the opinions across both types of devices, results show that the panel attributed a common relevance level for 16 aspects, five ‘Critical’ (two agreed by all stakeholders’ groups) and 11 ‘Fundamental’ (one agreed by all groups) (see Table 3). Examples of this are the ‘Clinical efficacy and/or effectiveness’ considered ‘Critical’ (majority in all groups), or the ‘Comfort for the patient’ considered ‘Fundamental’ (not getting majority in all groups but gathering a panel majority opinion). The former aspect is aligned with economic evaluation literature [6] centred into the effectiveness and costs of technologies whereas the latter is not explicitly considered in such methods. Moreover, many other aspects were recognised as relevant by the participants of our study, suggesting the need to formally consider a larger number of aspects in the evaluation of medical devices. This need has been recognised in literature [3, 47], by authors advocating for the use of MCDA in HTA [2], such as the ISPOR (The Professional Society for Health Economics and Outcomes Research) Medical Devices and Diagnostics Special Interest Group [48], by authors developing value framework models for evaluating medical devices, such as in the HTA Core Model from EUnetHTA (European network for Health Technology Assessment) [49], and also by the review on value assessment frameworks of Zhang et al. [22] that covered 19 studies addressing health technologies in general and 38 addressing specific types of health technologies (mainly drugs). Four of the frameworks reported in that review targeted diagnostic or genetic tests and one targeted nondrug health technologies, with evaluation aspects included varying between three and 16 and covering different devices’ features, namely, their medical benefit, the adverse effects, the quality of life and satisfaction of the patient, and the costs. Our study, besides validating this need with a large and diverse panel of stakeholders, adds additional value aspects not identified in the existing frameworks’ literature, e.g., regarding environmental impact and aspects related with devices’ usage by the healthcare professional, such as user-friendliness, the learning curve, the training and the workload.

The list of aspects included in our study tries to be purposefully inclusive, which brings the advantage of being as complete as possible but the disadvantage of entailing potential overlap in some aspects. To evolve towards the construction of a multidimensional framework or of multicriteria models, the identified aspects would require further work and restructuring, eventually combining and clarifying aspects and exploring how to measure them in practice [32] (for e.g., understanding what participants have in mind when considering the sensitivity and specificity of an implantable medical device as relevant). Nonetheless, our work provides important insights to inform such a framework development. In 44 frameworks reviewed by Zhang et al. [22], value aspects were identified through literature review, engagement of stakeholders, or a combination of both, but only four frameworks involved patients or citizens in aspects’ identification. Our study explored a way to collect the wide range and diversity of stakeholders’ perspectives, including patients and citizens, adding to the discussion on how to bring these insights into HTA for standardising and bringing guidance and transparency to the evaluation of medical devices [19, 28, 50] and how to include stakeholders’ views to inform HTA and adoption decisions [23, 26].

Methodologically, through the Web-Delphi it was thus possible, first, to involve a large and heterogeneous group of HTA stakeholders, enabling them to interact and learn with each other by sharing their views and build an agreement about the relevance of most aspects. Second, to draw conclusions about differences in opinion between stakeholders and across types of devices. Third, it has shown in which aspects there is a panel majority opinion. All of this provides input information for additional research on how to develop multidimensional evaluation models and frameworks, and assists in planning future directions of research.

### Implications for policy

This study shows that it is possible to gather the views of distinct stakeholders’ groups in a structured format, producing results that can be more widely used within HTA processes, as deemed as relevant by several authors [24–27, 51]. All aspects were considered to some extent relevant, and some aspects gathered the same relevance level irrespectively of the type of medical device under analysis. Accordingly, approaches to assess medical devices value need to consider a broader range of aspects and the specificities of distinct types of devices. Despite the heterogeneity of this type of technologies [12], there seems to be possible to attain some systematization and common standards, so asked in literature [4, 9, 20, 52]. Nevertheless, one should recall that the evaluation may be affected by the context [53], and that ‘Implantable medical devices’ and ‘In vitro tests based on biomarkers’ still comprise diverse devices, which needs to be considered when interpreting results.

### Limitations of the study

Several limitations should be acknowledged in this study. Firstly, this study takes place in Portugal, having only national participants, and thus results can be context- and/or country-dependent. Nevertheless, the list of aspects was based on international peer-reviewed literature which brings useful information to inform the discussion on HTA for medical devices, beyond the considered country and context. Secondly, as the Delphi process is highly dependent on the availability and commitment of participants [54], there was not a balance of participants across stakeholder groups, with the Buyers and policymakers and the Industry having a lower representation. This unbalance is somehow usual in Delphi processes as panels are purposive or convenience samples, not aiming to be representative samples of populations [54]. Furthermore, Delphi literature does not present unequivocal recommendations for the sample size, with studies suggesting ranges from five to more than one thousand participants [54, 55]. To try to mitigate the unbalance as much as possible, invitations and reminders for participation were sent. Thirdly, and still related with the Delphi process, it is important to acknowledge shortcomings of the method, namely the possibility of occurring cognitive biases and other behavioural influences during the process (such as egocentric discounting or the influence of majority positions) due to the freely online interactions among participants, which can also lead to answers not completely clarified by participants [56], or the possibility of information overload due to the high number of aspects to be analysed by the participants, which could become tiresome and cognitively challenging for them [57]. To avoid the occurrence of such shortcomings, not only the panel of participants was heterogeneous but also the aspects were organised, during the validation with experts, so that it would be easier, to the best of their knowledge, to follow the exercise. Additionally, participants could also rate each type of medical device in different time periods by re-accessing the platform, or even answering only one type of medical device if they felt more comfortable. Finally, the initial list of aspects could be biased as it was the result of a literature search followed by the validation by experts of the HTA agency. To overcome this possibility, participants could suggest additional aspects during the process, through the comments option, which was not observed.

## Conclusion

One hundred thirty-four participants, belonging to different groups of health stakeholders, recognised many aspects, besides costs and effectiveness, as relevant for the evaluation of ‘implantable’ and ‘in vitro’ medical devices. Results suggest the need to formally consider a larger number of aspects in such evaluations. The results from this study have implications for the development of multidimensional value frameworks and models in HTA for medical devices, contributing to guide evidence collection to inform evaluators. In future research, these results could be discussed by HTA agencies and decision-makers, namely for understanding the extent to which these findings can be applied for other types of medical devices and embedded within HTA processes. Additionally, research has been conducted within the scope of the MEDI-VALUE project and confirmed that the results of this study are a useful starting point for the development of multicriteria models to evaluate specific medical devices in real contexts, but this should be extended to other real-world settings.

## Supplementary Information

**Additional file 1: Table 1.1.** List and descriptions of the 34 aspects used in the Web-Delphi process, translated to English (original list and descriptions delivered in Portuguese).

**Additional file 2: Table 2.1.** Statistical tests results for each type of medical devices: Kruskal-Wallis with 4 degrees of freedom and Dunn post hoc test, corrected with Bonferroni test.

**Additional file 3: Table 3.1.** Percent agreement, Gwet’s coefficient of groups of stakeholders for both types of medical devices in both rounds of the Web-Delphi process, and strength of agreement according to Landis and Koch and to Gwet’s proposed benchmarking method.

## Data Availability

All data generated or analysed during this study are included within the article and its additional files.
