# Supplementary material for: Which value aspects are relevant for the evaluation of medical devices? Exploring stakeholders’ views through a Web-Delphi process
Source: BMC Health Serv Res. 2023 Jun 8;23:593. doi: 10.1186/s12913-023-09550-0 (PMC10249179; doi:10.1186/s12913-023-09550-0)
Supplement: Supplementary file 1 — Additional file 1: Table 1.1. List and descriptions of the 34 aspects used in the Web-Delphi process, translated to English (original list and descriptions delivered in Portuguese). [file 12913_2023_9550_MOESM1_ESM.pdf]

Additional file 1

Table 1.1. List and descriptions of the 34 aspects used in the Web-Delphi process, translated to English (original list and descriptions delivered in Portuguese).

**Aspect**	**Description**
Specific features of the medical device	Relevant characteristics such as: lifetime of the medical device, contact with the patient's body (direct: invasive and non-invasive; indirect), length of hospital stay; limitations compared to alternatives.
Technical performance of the medical device	Performance of a medical device or response of the patient(s) to the medical device regarding its intended purpose and as indicated by the manufacturer, when properly applied to the appropriate patient(s).
Regulatory status of the medical device	Current regulatory status of the medical device and adherence to market approval requirements.
Sensitivity and Specificity	Ability of the medical device to correctly identify positives and negatives in testing a population.
User-friendliness for the healthcare professional	Extent to which the medical device is comfortable and easy to be used by the healthcare professional (e.g., depth perception, eye-hand coordination or hours required to perform the examinations (compared to the expected time)]. The procedures for using the medical device are defined and clear. Extent to which the result of the medical device is easy to understand and interpret.
Time between procedure and results	Extent to which medical device results are instantly available.
Need for training of the healthcare professional	Need for training of health professionals to ensure specific knowledge necessary for the use of the medical device.
Learning curve of the healthcare professional	Improvements in the use of the medical device by the healthcare professional (e.g., evolution of speed and accuracy of use).
Exposure of the healthcare professional to physical or chemical agents	Exposure (internal or external) of the health professional to specific physical and chemical agents, metabolites, or reaction products.
Workload for the healthcare professional	Extent to which the medical device can be implemented without drastic changes in the current way of working and in the workload of healthcare professionals.
Comfort for the patient	Extent to which the use of the medical device is expected to improve the service offered to the patient (including patient satisfaction).
Connectivity	Ability to transfer information between different devices and decision support, including security solutions in the transmission of biosignals.
Clinical efficacy and/or effectiveness	Measure of the beneficial effect of a technology, i.e., the medical device's ability to produce a desired (beneficial) change in the signs, symptoms and evolution of the target health condition.
Risk analysis	Considers risk factors related to the potential number of patients at risk of harm: the likelihood of a medical device having problems, the likelihood of a patient being harmed, and the total number of patients exposed.
Adverse events for the patient	Severity and frequency of adverse events and their impacts.

Quality of the available scientific evidence	Robustness of the sources of evidence and quality of the evidence, regarding the target population and the intended clinical course, in a complete, consistent and relevant way, with the problems properly reported (risk of bias, imprecision, inconsistency, indirect evidence and publication bias).
Target population	Number of people affected by the target condition or relevant subgroup(s) in a specific time period.
Impact of the disease	Impact of health condition on: the disease burden for the patient and society, the incidence and prevalence and the natural history of the disease without treatment.
Patient-reported outcomes	Patient's perspective on a disease/treatment that may not be captured by a clinical measure, but of importance to the patient and their adherence to treatment or use of the medical device.
Quality of life for the patient	Expected impact on the patient's quality of life.
Space for innovation for the healthcare organization	Capacity to adopt the medical device.
Clinical guidelines	Extent to which the medical device is recommended in national clinical guidelines.
Financing	Extent to which the medical device is reimbursed; DRG (diagnostic-related groups) tariff or additional payments for the medical device (coverage).
Public health interest	Impact of the use of the medical device on public health and society.
Budget impact to the health system	Impact of medical device adoption on health system budget.
Equity	Fairness in the allocation of resources, treatments or results between different individuals or groups.
Market competitiveness	Competition in the market, in terms of the number of competing products.
Medical or technical complications for the patient	Expected occurrence and severity of medical and technical complications related to the procedure (for example, with surgery to implant the medical device).
Stakeholders agreement on the adoption of the medical device	Agreement between key actors (groups of stakeholders) or individuals in the health context, that is, the extent to which the adoption of the medical device is aligned with common goals.
Environmental impact of the production and use of the medical device	Extent to which the production and use or implementation of the medical device causes environmental damage.
Efficiency	Potential cost savings with the adoption of the medical device, considering the affordability of the medical device and the opportunity cost (lost alternatives).
Capacity of the health system	Adjustment of the medical device to the existing infrastructures and qualifications in the health system.
Cost of the medical device (including complementary equipment)	Costs with the medical device (e.g., disposable items) and other equipment inherent to the use of the medical device, maintenance (sterilization, licensing or updates) and training.
Cost of procedure without the cost of the medical device	Costs arising from the number of human resources required, time of use and infrastructure, for example, operating room (without considering the cost of the medical device).
