# Supplementary material for: Which value aspects are relevant for the evaluation of medical devices? Exploring stakeholders’ views through a Web-Delphi process
Source: BMC Health Serv Res. 2023 Jun 8;23:593. doi: 10.1186/s12913-023-09550-0 (PMC10249179; doi:10.1186/s12913-023-09550-0)
Supplement: Supplementary file 2 — Additional file 2: Table 2.1. Statistical tests results for each type of medical devices: Kruskal-Wallis with 4 degrees of freedom and Dunn post hoc test, corrected with Bonferroni test. [file 12913_2023_9550_MOESM2_ESM.pdf]

Additional file 2

Table 2.1. Statistical tests results for each type of medical devices: Kruskal-Wallis with 4 degrees of freedom and Dunn post hoc test, corrected with Bonferroni test.

	**Implantable medical devices**			**In vitro tests based on biomarkers**		
	**Kruskal-Wallis test**		**Dunn-Bonferroni test**	**Kruskal-Wallis test**		**Dunn-Bonferroni test**
	**H(4)**	**p-value**	**Groups with statistically significant differences (adjusted p-value)**	**H(4)**	**p-value**	**Groups with statistically significant differences (adjusted p-value)**
Specific features of the medical device	1.1202	0.8911		7.01	0.1354	
Technical performance of the medical device	3.255	0.5161		2.033	0.7297	
Regulatory status of the medical device	6.3812	0.1724		6.2413	0.1818	
Sensitivity and Specificity	15.447	0.003858*	Industry and Patients and citizens (0.0449*)	6.1482	0.1883	
User-friendliness for the healthcare professional	4	0.406		8.6689	0.06993	
Time between procedure and results	2.4344	0.6564		1.0451	0.9029	
Need for training of the healthcare professional	2.6251	0.6224		5.5108	0.2388	
Learning curve of the healthcare professional	9.4885	0.04998		3.3174	0.5062	
Exposure of the healthcare professional to physical or chemical agents	12.934	0.0116*	Healthcare professionals and Patients and citizens (0.0405*)	6.5662	0.1607	
Workload for the healthcare professional	2.7791	0.5955		7.9821	0.09224	
Comfort for the patient	5.4843	0.2411		6.6431	0.156	
Connectivity	4.0918	0.3937		1.6997	0.7908	
Clinical efficacy and/or effectiveness	3.8628	0.4249		4.9357	0.294	
Risk analysis	2.0747	0.722		2.797	0.5923	
Adverse events for the patient	3.5126	0.476		5.4578	0.2435	
Quality of the available scientific evidence	7.319	0.12		4.4289	0.3511	
Target population	11.702	0.01971*	Academics and Patients and citizens (0.0447*)	9.5509	0.04871*	Academics and Patients and citizens (0.0370*)
Impact of the disease	9.8507	0.04302*	Industry and Patients and citizens (0.0282*)	5.5522	0.2352	
Patient-reported outcomes	7.2508	0.1232		4.2598	0.372	
Quality of life for the patient	6.8498	0.144		6.2529	0.181	
Space for innovation for the healthcare organization	4.5155	0.3407		3.4667	0.483	
Clinical guidelines	1.5567	0.8166		4.1905	0.3808	
Financing	4.4924	0.3435		5.0755	0.2796	

Public health interest	8.971	0.06183		3.8203	0.4309	
Budget impact to the health system	2.9802	0.5611		8.8985	0.06369	
Equity	4.189	0.381		1.7564	0.7804	
Market competitiveness	8.5699	0.0728		2.0077	0.7343	
Medical or technical complications for the patient	4.5029	0.3422		1.8812	0.7576	
Stakeholders agreement on the adoption of the medical device	2.1309	0.7117		1.1193	0.8912	
Environmental impact of the production and use of the medical device	5.4038	0.2483		0.82552	0.935	
Efficiency	7.2125	0.1251		4.5049	0.342	
Capacity of the health system	4.1637	0.3843		1.9728	0.7408	
Cost of the medical device (including complementary equipment)	11.058	0.02592*	Academics and Patients and citizens (0.0266*)	17.322	0.001674*	Academics and Healthcare professionals (0.00718*)
Cost of procedure without the cost of the medical device	12.119	0.01649*	Academics and Healthcare professionals (0.0326*)	7.5593	0.1091	

* p-value < 0.05
