# Supplementary material for: Which value aspects are relevant for the evaluation of medical devices? Exploring stakeholders’ views through a Web-Delphi process
Source: BMC Health Serv Res. 2023 Jun 8;23:593. doi: 10.1186/s12913-023-09550-0 (PMC10249179; doi:10.1186/s12913-023-09550-0)
Supplement: Supplementary file 3 — Additional file 3: Table 3.1. Percent agreement, Gwet’s coefficient of groups of stakeholders for both types of medical devices in both rounds of the Web-Delphi process, and strength of agreement according to Landis and Koch and to Gwet’s proposed benchmarking method. [file 12913_2023_9550_MOESM3_ESM.pdf]

Additional file 3

Table 3.1. Percent agreement, Gwet's coefficient of groups of stakeholders for both types of medical devices in both rounds of the Web-Delphi process, and strength of agreement according to Landis and Koch and to Gwet's proposed benchmarking method.

Groups of stakeholders	Round 1				Round 2			
	Percent agreement	Gwet's AC2 (95% CI)	Landis and Koch's strength of agreement	Gwet's strength of agreement[1]	Percent agreement	Gwet's AC2 (95% CI)	Landis and Koch's strength of agreement	Gwet's strength of agreement[1]
1MPLANTABLE MEDICAL DEVICES								
Academics	0.88	0.68 (0.62 – 0.74)	Substantial	Substantial	0.90	0.74 (0.69 – 0.79)	Substantial	Substantial
Buyers and policymakers	0.88	0.65 (0.57 – 0.73)	Substantial	Moderate	0.91	0.76 (0.69 – 0.83)	Substantial	Substantial
Healthcare professionals	0.90	0.73 (0.70 – 0.76)	Substantial	Substantial	0.91	0.77 (0.75 – 0.80)	Substantial	Substantial
Industry	0.89	0.70 (0.63 – 0.76)	Substantial	Substantial	0.90	0.73 (0.67 – 0.79)	Substantial	Substantial
Patients and citizens	0.89	0.70 (0.67 – 0.74)	Substantial	Substantial	0.92	0.79 (0.75 – 0.82)	Substantial	Substantial
IN VITRO TESTS BASED ON BIOMARKERS								
Academics	0.88	0.65 (0.60 – 0.71)	Substantial	Substantial	0.91	0.75 (0.71 – 0.79)	Substantial	Substantial
Buyers and policymakers	0.85	0.55 (0.41 – 0.69)	Moderate	Moderate	0.88	0.67 (0.56 – 0.78)	Substantial	Moderate
Healthcare professionals	0.89	0.69 (0.66 – 0.73)	Substantial	Substantial	0.90	0.74 (0.70 – 0.77)	Substantial	Substantial
Industry	0.84	0.52 (0.42 – 0.62)	Moderate	Moderate	0.85	0.56 (0.47 – 0.65)	Moderate	Moderate
Patients and citizens	0.87	0.63 (0.59 – 0.66)	Substantial	Substantial	0.89	0.70 (0.66 – 0.74)	Substantial	Substantial

[1]The strength of agreement was established following Gwet's proposed benchmarking method, with the Landis and Koch benchmark scale and the 95% cut-off point for the cumulative probabilities.
